# Multiparametric MRI for Characterization of the Basal Ganglia and the Midbrain

**DOI:** 10.3389/fnins.2021.661504

**Published:** 2021-06-21

**Authors:** Till M. Schneider, Jackie Ma, Patrick Wagner, Nicolas Behl, Armin M. Nagel, Mark E. Ladd, Sabine Heiland, Martin Bendszus, Sina Straub

**Affiliations:** ^1^Department of Neuroradiology, University of Heidelberg, Heidelberg, Germany; ^2^Department of Artificial Intelligence, Fraunhofer Heinrich Hertz Institute, Berlin, Germany; ^3^Division of Medical Physics in Radiology, German Cancer Research Center, Heidelberg, Germany; ^4^Institute of Radiology, University Hospital Erlangen, Friedrich-Alexander-Universität Erlangen-Nürnberg, Erlangen, Germany; ^5^Faculty of Physics and Astronomy and Faculty of Medicine, University of Heidelberg, Heidelberg, Germany

**Keywords:** quantitative susceptibility mapping, machine learning, magnetization transfer, basal ganglia, magnetic resonance imaging, ultra high field, sodium imaging

## Abstract

**Objectives** To characterize subcortical nuclei by multi-parametric quantitative magnetic resonance imaging.

**Materials and Methods:** The following quantitative multiparametric MR data of five healthy volunteers were acquired on a 7T MRI system: 3D gradient echo (GRE) data for the calculation of quantitative susceptibility maps (QSM), GRE sequences with and without off-resonant magnetic transfer pulse for magnetization transfer ratio (MTR) calculation, a magnetization−prepared 2 rapid acquisition gradient echo sequence for T_1_ mapping, and (after a coil change) a density-adapted 3D radial pulse sequence for ^23^Na imaging. First, all data were co-registered to the GRE data, volumes of interest (VOIs) for 21 subcortical structures were drawn manually for each volunteer, and a combined voxel-wise analysis of the four MR contrasts (QSM, MTR, T_1_, ^23^Na) in each structure was conducted to assess the quantitative, MR value-based differentiability of structures. Second, a machine learning algorithm based on random forests was trained to automatically classify the groups of multi-parametric voxel values from each VOI according to their association to one of the 21 subcortical structures.

**Results** The analysis of the integrated multimodal visualization of quantitative MR values in each structure yielded a successful classification among nuclei of the ascending reticular activation system (ARAS), the limbic system and the extrapyramidal system, while classification among (epi-)thalamic nuclei was less successful. The machine learning-based approach facilitated quantitative MR value-based structure classification especially in the group of extrapyramidal nuclei and reached an overall accuracy of 85% regarding all selected nuclei.

**Conclusion** Multimodal quantitative MR enabled excellent differentiation of a wide spectrum of subcortical nuclei with reasonable accuracy and may thus enable sensitive detection of disease and nucleus-specific MR-based contrast alterations in the future.

## Introduction

Subcortical nuclei of the basal ganglia, midbrain and brainstem are interconnected structures of gray matter that play an instrumental role in the integration of motor as well as non-motor behavioral functions of the brain (Nelson and Kreitzer, 2014; Simonyan, 2019). In recent years, subcortical nuclei have not only been implicated in the pathophysiology of a wide range of motor function affecting diseases such as Parkinson’s disease (Obeso et al., 2008) or atypical Parkinsonian syndromes (Broski et al., 2014; Saeed et al., 2017), but also in neurodegenerative diseases such as Alzheimer’s disease (Arendt et al., 2015; Eser et al., 2018), Huntington’s disease (Eser et al., 2018) and frontotemporal lobar degeneration (Looi et al., 2008; Halabi et al., 2013) or in non-degenerative neuropsychiatric conditions such as obsessive-compulsive disorders, depression or chronic pain (Lacerda et al., 2003; Bielau et al., 2005; Kumar et al., 2014; Taylor and Westlund, 2017; Zhang et al., 2019). In search of sensitive biomarkers for subcortical cerebral diseases, magnetic resonance imaging (MR)-based studies have long focused on the assessment of morphological subcortical changes quantified by voxel-based morphometry (VBM) using structural data only. In recent years, quantitative MR-based techniques such as diffusion tensor imaging (DTI), quantitative susceptibility mapping (QSM), magnetization transfer ratio (MTR) or sodium imaging have additionally shown great potential for the assessment of subcortical structures especially at ultra-high field strength (Reetz et al., 2012; Tambasco et al., 2015; Andica et al., 2019; Mazzucchi et al., 2019). However, as diseases often affect multiple subcortical nuclei to a varying extent, e.g., by protein deposition in several subcortical structures (Dugger and Dickson, 2017), a lack of specificity still challenges MR-based classification of diseases on the basis of single MR contrasts or single subcortical structures (Saeed et al., 2017) and a characteristic, multiparametric MR-based footprint of healthy subcortical structures may be a requisite for future MR-based discrimination of subcortical diseases.

The present study assesses subcortical nuclei of the basal ganglia and the midbrain using QSM, MTR, sodium imaging and T_1_ relaxation time mapping at 7T. QSM not only provides an excellent image contrast for optimized discrimination of basal ganglia (Deistung et al., 2013a; Keuken et al., 2014), but also enables detection of increased iron deposition in the basal ganglia associated with a range of degenerative and inflammatory diseases such as multiple sclerosis, Parkinson’s and Huntington’s disease as well as alcohol use disorder (Wallis et al., 2008; Dominguez et al., 2016; Langkammer et al., 2016; Juhas et al., 2017; Zivadinov et al., 2018). Similarly, increased ^23^Na concentrations in cerebral gray matter have been linked to Alzheimer’s disease and an increasing severity of multiple sclerosis (Mellon et al., 2009; Zaaraoui et al., 2012), as ^23^Na concentrations are presumably dependent on the volume of extracellular space and cellular membrane integrity (O’Brien et al., 1974; Boada et al., 2005). Finally, reduced subcortical T_1_ relaxation times have been associated with gray matter loss following neurodegenerative disease (Baudrexel et al., 2010), and MTR imaging of subcortical structures has shown promising results for the discrimination of Parkinson’s disease and atypical Parkinson syndromes (Eckert et al., 2004) as it uses radiofrequency off-resonance pulses to saturate macromolecule-associated protons. The resulting magnetization transfer is dependent on the exchange rate between pools of coupled and free protons and correlates with the concentration of macromolecules (Wolff and Balaban, 1994; Henkelman et al., 2001; Horsfield et al., 2003; Peper et al., 2013).

The aim of this study is the investigation of a novel, multiparametric approach to characterize subcortical nuclei based on the assessment of combined voxel-intrinsic MR values from four different quantitative MR contrasts (QSM, MTR, T_1_, ^23^Na) in healthy volunteers.

## Materials and Methods

### Data Acquisition

The study was conducted in accordance with the Declaration of Helsinki. Institutional review board approval was obtained and all subjects provided written informed consent. Five healthy volunteers (mean age 28.4 ± 6.5 years; three female) underwent MRI on a 7T whole-body system (MAGNETOM 7T, Siemens Healthcare GmbH, Germany). At the beginning of each imaging session and after the coil change, *B*_0_ shimming was performed using the vendors’ default second-order routines. For all ^1^H scans, *B*_1_-calibration was performed with a pre-saturation-based 2D turbo flash sequence and for sodium imaging by measuring the total ^23^Na signal intensity as a function of transmitter voltage. A monopolar 3D gradient echo (GRE) sequence for susceptibility mapping, two vendor−provided 2D proton density (PD)-weighted GRE sequences with and without additional off-resonant MT-pulse (500°, 1.5 kHz off-resonance, 7.68 ms duration) for MTR calculation, and a magnetization−prepared 2 rapid acquisition gradient echo (MP2RAGE) sequence with inversion times TI_1_ = 900 ms, TI_2_ = 2,700 ms and online vendor-provided T_1_ map calculation were acquired with a 8Tx/32Rx-channel head coil (Nova Medical Inc., Wakefield, MA, United States) operated using an in-house-built Butler matrix with sequence parameters given in Table 1. After a coil change, ^23^Na data were acquired using a double-resonant ^1^H/^23^Na Tx/Rx quadrature volume head coil integrating a 30-channel ^23^Na Rx phased array (Rapid Biomedical GmbH, Rimpar, Germany) and a density adapted 3D radial pulse sequence with TR = 100 ms, TE_1_ = 0.35 ms, flip angle = 90°, 2.0 mm nominal isotropic resolution, 7,000 projections, TA = 11:40 min (Nagel et al., 2009), and a T_1_-weighted GRE sequence was acquired to facilitate image registration.

**TABLE 1:** Sequence parameters including voxel size, matrix size, repetition time (TR), echo time (TE), flip angle (FA), bandwidth (BW), parallel imaging (GRAPPA), and partial Fourier specifics and acquisition time (TA).

	**Resolution (mm^3^), matrix size**	**TR (ms)**	**TE (ms)**	**FA (°)**	**BW (Hz/px)**	**GRAPPA (Griswold et al., 2002) (factor/ref. lines)**	**Partial Fourier (slice, phase)**	**TA (min:sec)**
ME-GRE	0.5 × 0.5 × 0.5, 448 × 336 × 224	21	6/12/18	10	490	2/36	7/8, 6/8	11:23
GRE+/−MT	0.7 × 0.7 × 2.0, 320 × 240 × 36	197	3.47	8	220	3/36	6/8, 6/8	9:25
MP2RAGE	0.7 × 0.7 × 0.7, 320 × 240 × 208	5,000	3.63	4,5	290	3/48	−, 6/8	8:02

### Data Processing

Susceptibility maps were generated from phase data that were combined on the scanner using ASPIRE (Eckstein et al., 2018). Brain masks were calculated using FSL-BET (Smith, 2002) from the first echo of the GRE magnitude data. Laplacian-based phase unwrapping, V-SHARP (Li et al., 2011, 2014; Wu et al., 2012) with kernel size up to 12 mm for background field removal and STAR-QSM (Wei et al., 2015) were used in Matlab (MathWorks, Natick, United States) to calculate susceptibility maps. ^23^Na data were reconstructed using an in−house Matlab tool with adaptive combination (Benkhedah et al., 2016). Correction of the receive profile was performed using the transmit/receive birdcage coil located around the receive array. Sodium data were referenced such that mean cerebrospinal fluid (CSF) sodium values equaled the physiological concentration of 140 mmol/l. Based on the recorded T_1_-weighted images, sodium data were registered to QSM in the Medical Imaging Interaction Toolkit (MITK) (Maleike et al., 2009; Nolden et al., 2013). Semi-quantitative MTR maps were calculated in Matlab dividing the difference between the non-MT-saturated and the MT-saturated data by non-MT-saturated data and multiplied by 100. To define the volumes of interest (VOIs) for subcortical nuclei a neuroradiologist (TS) with 8 years of experience and special expertise in the assessment of deep gray matter nuclei used susceptibility maps superimposed on MT-saturated PD images in MITK to manually segment VOIs for each volunteer.

### Functional Group Definition of Subcortical Nuclei and Fiber Tracts

**FIGURE 1:**
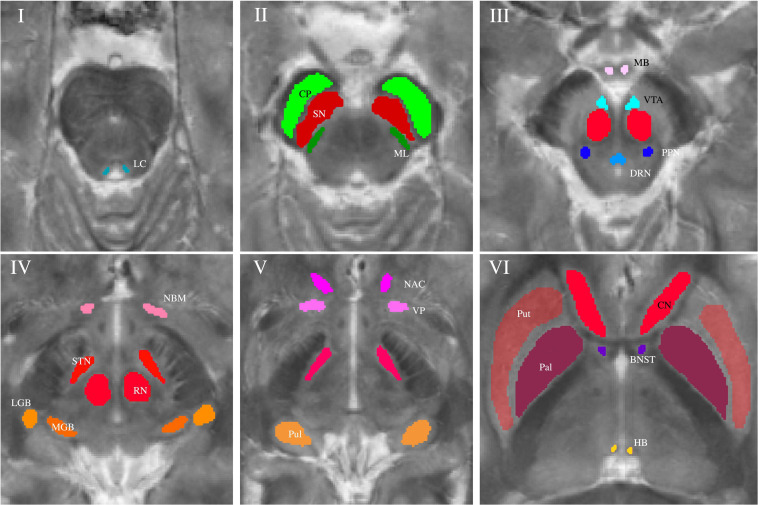
Volumes of interest (VOIs) are indicated on axial QSM/MT overlay images numerated from caudally to cranially in ascending order. VOIs of fiber tracts (CP and ML) are colored in shades of green in Slice II. VOIs of (epi-)thalamic nuclei (LGB, MGB, HB, and Pul) are colored in shades of yellow in Slices IV and V, VOIs of limbic nuclei (BNST, NAC, VP, MB, and NBM) are colored in shades of pink in Slices III-VI and, VOIs of nuclei of the ARAS (DTN, PPN, VTA, and LC) in shades of blue in Slices I and III. VOIs of extrapyramidal nuclei (NC, RN, PAL, Put, SN, and STN) are colored in shades of red in Slices II to VI.

Subcortical nuclei and fiber tracts were grouped into five groups and are shown in Figure 1. The group of (epi-)thalamic nuclei consists of the medial and lateral geniculate bodies (MGB and LGB), the pulvinar (Pul) and the habenula (HB). Nuclei associated with the ascending reticular activating system (ARAS) are the ventral tegmental area (VTA), the pedunculopontine nucleus (PPN), the locus coeruleus (LC), and the dorsal raphe nucleus (DRN). The group of limbic nuclei includes the ventral pallidum (VP), the nucleus basalis Meynert (NBM), the bed nucleus of the stria terminalis (BNST), the nucleus accumbens (NAC), and the mamillary body (MB). The substantia nigra (SN), the subthalamic nucleus (STN), the red nucleus (RN), the globus pallidus (Pal), the putamen (Put), and the nucleus caudatus (NC) correspond to the group of extrapyramidal nuclei. The group of fiber tracts included the cerebral peduncle (CP) and the medial lemniscus (ML). Groups of nuclei and fiber tracts are summarized in Table 2.

**TABLE 2:** Groups of nuclei and fiber tracts with abbreviations.

**(Epi-)thalamic nuclei**	
Medial and lateral geniculate bodies	MGB and LGB
Pulvinar	Pul
Habenula	HB
**Ascending reticular activating system (ARAS)**	
Ventral tegmental area	VTA
Dorsal raphe nucleus	DRN
Pedunculopontine nucleus	PPN
Locus coeruleus	LC
**Limbic nuclei**	
Ventral pallidum	VP
Nucleus basalis Meynert	NBM
Bed nucleus of the stria terminalis	BNST
Nucleus accumbens	NAC
Mamillary body	MB
**Extrapyramidal nuclei**	
Substantia nigra	SN
Subthalamic nucleus	STN
Red nucleus	RN
Globus pallidus	Pal
Putamen	Put
Nucleus caudatus	NC
**Fiber tracts**	
Cerebral peduncle	CP
Medial lemniscus	ML

### Data Analysis

Mean and standard deviations were calculated for susceptibility, MTR, sodium concentration and T_1_ relaxation time measurements in each structure and a ranking of mean values in ascending order was established for each of the four contrasts. A voxel-wise correlation analysis between each of the different MR contrasts indifferent of subcortical structures was conducted calculating Pearson’s correlation coefficient. *P*-values below 0.05 were considered statistically significant.

Four-dimensional (three axes for T1 values, MT ratio, and susceptibility values; the fourth dimension for sodium concentration is represented by color) scatter plots were generated for each of the groups of nuclei combining a visualization of the four contrasts in each voxel. For each subcortical nucleus tri-axial ellipsoids were centered at the mean signal intensity in each contrast and axial lengths of the ellipsoids correspond to the standard deviation regarding QSM, MTR and T_1_- measurements.

Furthermore, three prediction tasks were solved based on random forest analyses (Breiman, 2001) (Python 3.6, scikit-learn 0.23.1) to assess machine learning-based classification of the different subcortical structures on the basis of multi-parametric voxel-values from each VOI, represented by matrices containing voxel-values only. Nuclei from the same subject were considered as independent samples. Firstly, to predict the subcortical structure to which the combined quantitative values from a VOI belong, secondly, to predict the functional group of each VOI, and thirdly, to classify structures within each functional group. The significance of each MR contrast for each task was additionally analyzed using random forests with 100 independent random trees, equaling 100 classifiers. For the decision trees eight features for each of the four contrasts were defined: The voxel data was transformed into a one-dimensional signal. The mean value, the variance, the minimal value and the maximal value of each contrast and its gradient, respectively, were computed based on the flattened signal (32 features for each sample in total). These features are summarized in Table 3. The model prediction was evaluated using a leave-one-out cross-validation. To compensate for statistical variances that occurred due to using randomness-based methods, all reported results were averaged across 100 runs. Results were illustrated by normalized confusion matrices indicating predicted and true classes with the diagonal elements representing the probability of the predicted class being equal to the true class. Very small structures, namely, MB, the LC and the HB, were excluded from the automated analysis because of high partial volume effects due to the low resolution of sodium imaging. To evaluate the importance of multiparametric MRI for the described classification of the different subcortical structures on the basis of multi-parametric voxel-values from each VOI, a meta-experiment using only a single contrast or a subset of contrasts was conducted. Moreover, the segmentation performance for various machine learning methods and feasibility of discrimination of subcortical nuclei against the surrounding cerebral tissue was included in Supplementary Material.

**TABLE 3:** Description of features used for the random forest analysis.

**Feature per sequence QSM, Na, MTR, T_1_**	**Description**
mean_	Mean value of the given data
var_	Variance of the given data
min_	Minimal value of the given data
max_	Maximal value of the given data
mean_grad_	Mean value of the gradient of the given data
var_grad_	Variance of the gradient of the given data
min_grad_	Minimal value of the gradient of the given data
max_grad_	Maximal value of the gradient of the given data

## Results

### Descriptive and Statistical Analysis

**FIGURE 2:**
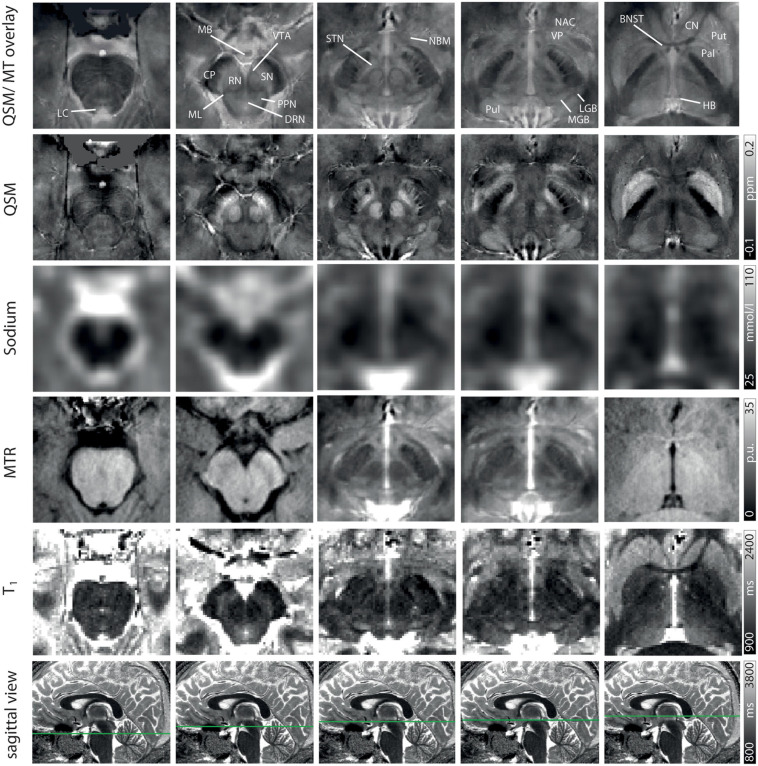
Representative axial slices at five indicated levels of the midbrain for a QSM/MT overlay, QSM, sodium images, MTR w, T_1_ from Row 1–5. In Row 6 the axial level of each row of images is indicated on sagittal T_1_-maps. Structures are indicated on the QSM/MT overlay. All data were co-registered to the gradient echo data which were used to calculate QSM.

Exemplary slices of the multi-parametric data including an overlay of QSM and MT-saturated PD are shown in Figure 2. QSM and the QSM/MT-overlay display the anatomical substructures of the basal ganglia and midbrain with the highest contrast, followed by T_1_ and MTR. In sodium imaging, Put and Pal can be visually differentiated. A slight increase in contrast can be visually appreciated for the BNST, PPN, DRN, and the ML on the QSM/MT-overlay compared to QSM contrast alone.

Table 4 shows the mean values and standard deviations of susceptibility, MTR, MR-based sodium concentration and T_1_ relaxation time measurements for each of the subcortical structures. Mean susceptibility across all studied subcortical nuclei was 0.031 ± 0.037 ppm, mean sodium concentration was 51.0 ± 14.4 mmol/l, mean MTR was 22.0 ± 2.0 percentage units (p.u.) and mean T_1_ time was 1448.5 ± 179 ms. Fiber tracts showed a mean susceptibility of −0.028 ± 0.007 ppm, a mean sodium concentration of 42.4 ± 3.5 mmol/l, a mean MTR of 24 ± 1 p.u. and a mean T_1_ time of 1,231 ± 83 ms.

**TABLE 4:** Mean susceptibility, sodium concentration, MTR and T_1_ values are given with standard deviation for each nucleus.

	**Susceptibiliy** χ **(ppm)**	**Sodium (mmol/l)**	**MTR (p.u.)**	**T_1_ (ms)**
Nucleus caudatus	0.0289 ± 0.0230	53.1 ± 4.2	19.4 ± 2.6	1,709 ± 44
Red nucleus	0.0651 ± 0.0153	38.9 ± 1.2	26.1 ± 0.6	1,167 ± 25
Globus pallidus	0.0851 ± 0.0095	38.8 ± 2.0	23.0 ± 1.7	1,237 ± 15
Putamen	0.0255 ± 0.0086	47.1 ± 2.9	18.5 ± 0.8	1,614 ± 40
Nucleus accumbens	−0.0148 ± 0.0144	48.5 ± 2.7	22.3 ± 1.9	1,602 ± 31
Locus coeruleus	0.0163 ± 0.0056	80.3 ± 7.3	20.2 ± 2.1	1,725 ± 79
Bed nucleus of stria terminalis	−0.0319 ± 0.0177	47.5 ± 4.8	21.0 ± 1.9	1,702 ± 54
Substantia nigra	0.0901 ± 0.0174	37.2 ± 2.8	23.3 ± 0.6	1,292 ± 16
Subtalamic nuclus	0.0747 ± 0.0188	35.2 ± 2.5	25.4 ± 1.3	1,141 ± 27
Ventral tegmental area	0.0228 ± 0.0120	43.0 ± 2.8	24.5 ± 0.8	1,308 ± 40
Cerebral peduncle	−0.0374 ± 0.0116	40.0 ± 3.7	25.2 ± 1.0	1,153 ± 38
Dorsal raphe nucleus	−0.0110 ± 0.0076	53.7 ± 3.6	22.0 ± 1.4	1,589 ± 63
Lateral geniculate body	0.0077 ± 0.0069	45.8 ± 3.7	19.2 ± 1.5	1,397 ± 48
Mammillary body	0.0341 ± 0.0097	64.6 ± 7.3	21.3 ± 2.0	1,477 ± 44
Medial geniculate body	0.0337 ± 0.0190	57.2 ± 5.5	21.3 ± 1.1	1,513 ± 44
Pedunculopontine nucleus	0.0021 ± 0.0097	46.4 ± 4.5	24.1 ± 1.2	1,370 ± 21
Pulvinar	0.0456 ± 0.0094	49.8 ± 4.7	20.6 ± 1.4	1,510 ± 44
Ventral pallidum	0.0943 ± 0.0151	40.9 ± 2.2	24.3 ± 3.0	1,270 ± 34
Habenula	0.0244 ± 0.0210	92.9 ± 6.8	24.0 ± 1.4	1,397 ± 53
Medial leminscus	−0.0208 ± 0.0056	46.4 ± 3.4	22.6 ± 1.5	1,312 ± 30
Nucleus basalis Meynert	−0.0180 ± 0.0184	46.7 ± 4.5	20.5 ± 3.3	1,487 ± 95

The results of the voxel-wise correlation analysis for each pair of MR contrasts showed a moderate positive correlation between T_1_ time and sodium concentration (*r*_Pearson_ = 0.58, *p* < 0.001) and a moderate negative correlation of T_1_ time with MTR (*r*_Pearson_ = −0.46, *p* < 0.001). Only minor negative correlations were found between sodium concentration and MTR (*r*_Pearson_ = −0.25, *p* < 0.001) and between T1 and QSM (*r*_Pearson_ = −0.24, *p <* 0.001). MTR and QSM showed no correlation at all (*r*_Pearson_ = −0.01, *p <* 0.001).

**FIGURE 3:**
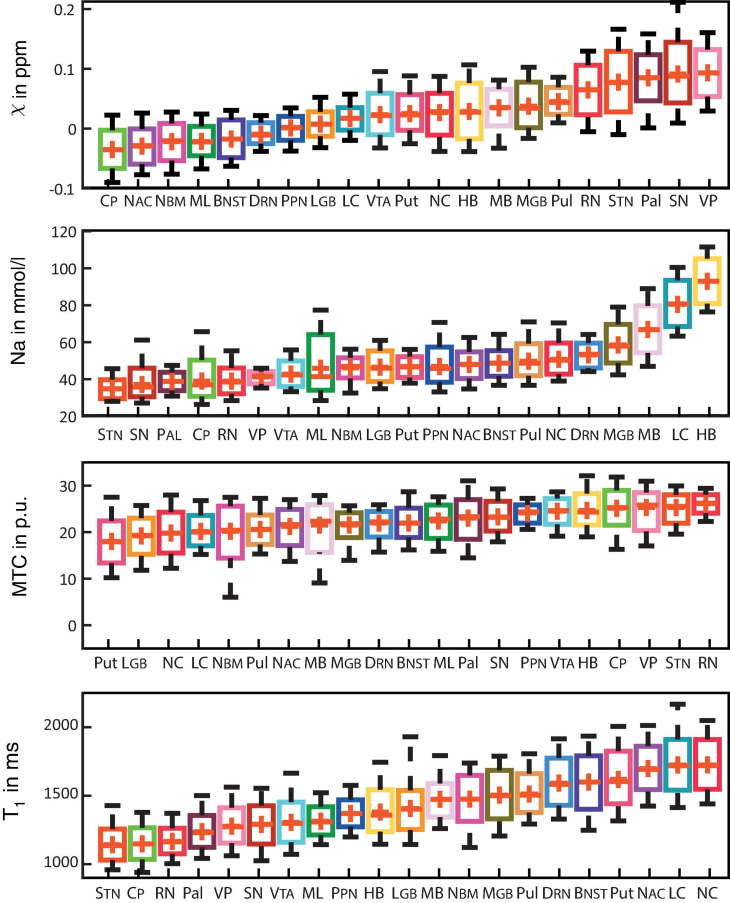
Boxplots for each of the recorded contrasts displaying the voxel-wise signal in each subcortical structure in ascending order and colored according to Figure 1. Additionally, mean values are indicated by a red cross for each structure and whiskers represent the 9th and 91st percentiles. (Epi-)thalamic nuclei including medial and lateral geniculate bodies (MGB and LGB), the pulvinar (Pul), and the habenula (HB) are colored in shades of yellow. Limbic nuclei including the ventral pallidum (VP), the nucleus basalis Meynert (NBM), the bed nucleus of the stria terminalis (BNST), the nucleus accumbens (NAC) and the mamillary body (MB) are colored in shades of pink. Nuclei of the ARAS including the ventral tegmental area (VTA), the pedunculopontine nucleus (PPN), the locus coeruleus (LC), and the dorsal raphe nucleus (DRN) are colored in shades of blue. Extrapyramidal nuclei including the substantia nigra (SN), the subthalamic nucleus (STN), the red nucleus (RN), the globus pallidus (Pal), the putamen (Put), and the nucleus caudatus (NC) are colored in shades of red. Fiber tracts including the cerebral peduncle (CP) and the medial lemniscus (ML) are colored in shades of green.

Figure 3 shows box plots of susceptibility values, sodium concentrations, MTR, and T_1_ times of all subcortical structures in ascending order. In the group of limbic nuclei, contrast characteristics of the VP stand out with relatively highest values for MTR and QSM and lowest values for sodium concentration and T_1_-times. With regard to QSM the NAC, NBM and BNST show similar values compared to the evaluated fiber tracts and display the lowest susceptibility values of all investigated subcortical nuclei. The subcortical structures of the ARAS are mostly distributed in the middle of the range of subcortical structures in each contrast and the extrapyramidal subcortical structures show particularly high susceptibility values while displaying notably low sodium concentration in most structures. Compared to the rest of the extrapyramidal nuclei, NC and Put are set apart by values at the opposite end of the range of mean MTR and T_1_ values and in the middle to upper range of subcortical structures regarding sodium concentration. High sodium levels are especially noted for MB, LC and HB. In relation to nuclei, the investigated fiber tracts showed low susceptibility values, intermediate to low T_1_ times and sodium concentrations and intermediate to high MTR values. These tendencies are more pronounced for the CP than for the ML in all studied contrasts.

**FIGURE 4:**
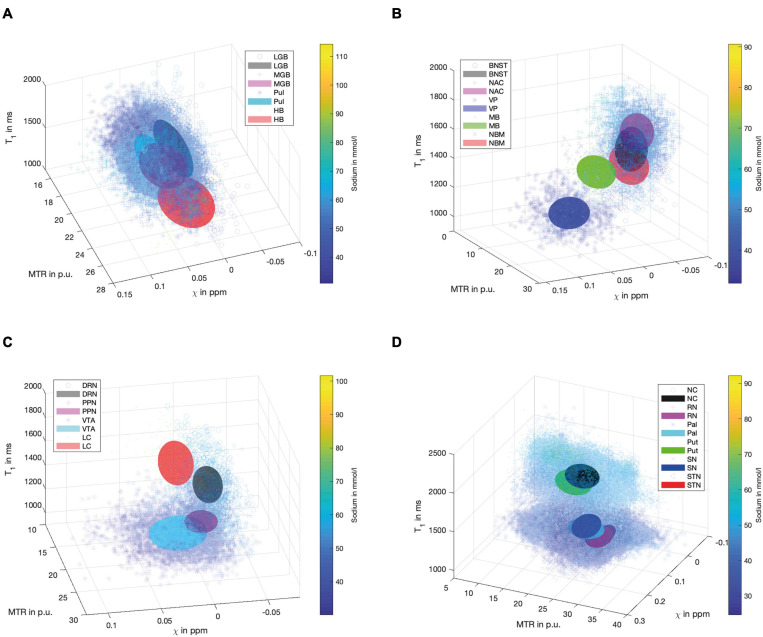
Scatter plots for the group of (epi-)thalamic nuclei **(A)** with the LGB (black), the MGB (magenta), the PUL (cyan) and the HB (red), the limbic nuclei **(B)** with the BNST (black), NAC (magenta), VP (blue), MB (green) and the NBM (red), the ARAS **(C)** with the DRN (black), PPN (magenta), VTA (cyan), and LC (red) and the extrapyramidal nuclei **(D)** with the NC (black), RN (magenta), PAL (cyan), PUT (green), SN (blue), and STN (red) are shown. Tri-axial ellipsoids are centered at the mean signal intensity of a subcortical nucleus in each imaging contrast and axial lengths of the ellipsoids correspond to the standard deviation in each contrast. Markers and colors of ellipsoids represent each structure according to the legends of **(A–D)**. Each marker is colored according to the voxel-wise sodium concentration.

Figure 4 demonstrates scatter plots of the different functional groups enabling a quantitative and integrated multimodal visualization of the four contrasts in each voxel within the subcortical structures. In the (epi-)thalamic group of nuclei (Figure 4A), the ellipsoids of the LGB, the PUL and the HB have no overlap, while the MGB reveals overlap with each of the other structures. The nuclei of the limbic system (Figure 4B) overlap with the ellipsoid of the BNST with the NBM and the NAC ellipsoids while the ellipsoids of the MB and the VP remain unaffected. With regard to the nuclei affiliated with the ARAS (Figure 4C), the ellipsoids of the DRN and the LC are well distinguishable while a small overlap is visible between ellipsoids of the PPN and the VTA. In the extrapyramidal system (Figure 4D), the ellipsoids of the NC and Put show a large overlap yet display higher sodium values and are distant to the remaining extrapyramidal nuclei. Further overlap is noted between the ellipsoids of the SN and PAL as well as between the RN and the STN while RN and SN ellipsoids show no overlap.

### Automated Signal-Based Characterization of Subcortical Structures

**FIGURE 5:**
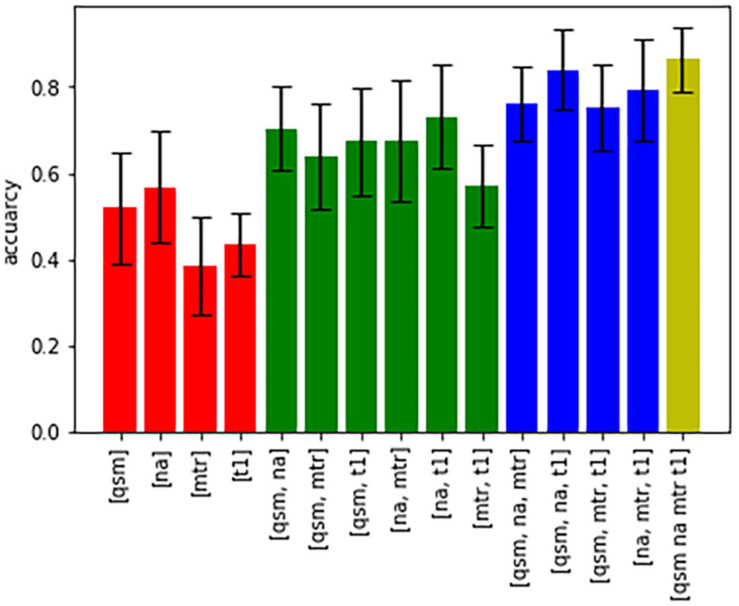
Bar graphs showing the accuracy of characterization of all nuclei based on different combinations of contrasts.

Figure 5 shows bar graphs displaying the accuracy (of 100 runs of five-fold cross-validation) for the characterization of subcortical structures, when only one, a combination of different contrasts, or all contrasts are used for classification. The highest accuracy was achieved when all four contrasts were available, otherwise an accuracy above 80% was only reached in the combination of QSM, sodium imaging and T_1_-times. The downstream performances increase as the number of different contrasts increases, which is a strong argument for the characterization of nuclei with multi-contrast MRI.

**FIGURE 6:**
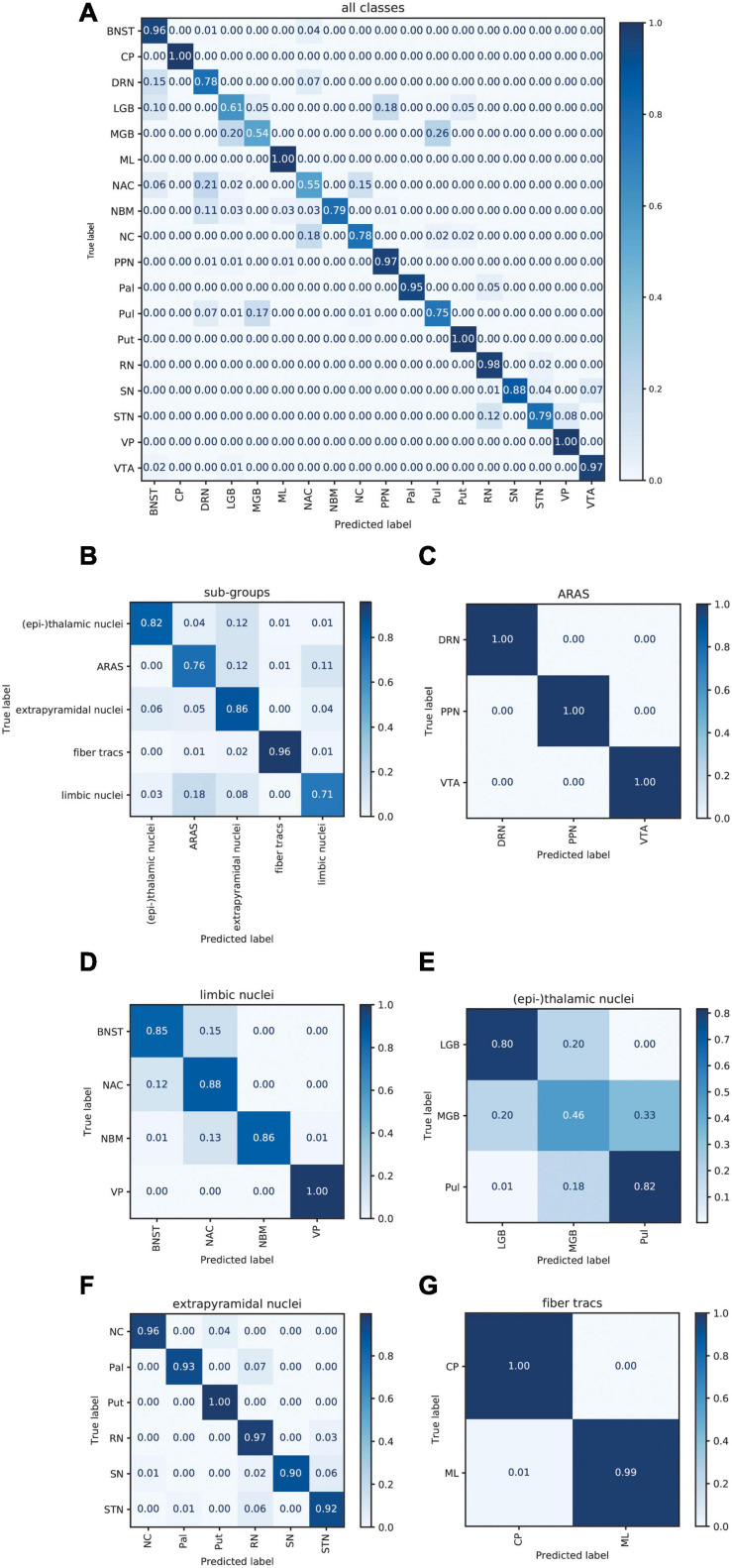
Normalized sum of confusion matrices of five patients (mean values of 100 runs) for the classification of all subcortical structures **(A)**, the classification into functional groups **(B)** and the discrimination of nuclei of the epi-thalamic group **(C)**, the limbic group **(D)**, the ARAS **(E)**, the extrapyramidal nuclei **(F)**, and the fiber tracts **(G)**. The diagonal elements represent the probability of the predicted class being equal to the true class in values between zero and one. Off-diagonal elements are those mislabeled by the classifier.

Figure 6A displays the average sum of confusion matrices of all five volunteers across 100 runs and for each investigated subgroup demonstrating the main confounders for each subcortical structure. Table 5 displays the importance of each feature in each contrast for the respective prediction task. The overall accuracy for correct classification of all subcortical structures based on the signal of all four contrasts was 85% (as a mean value of 100 runs) with the highest accuracy of correctly predicted classification of 100% achieved for Put and the lowest accuracies for the MGB, NAC, LGB, NC, and DRN with 54, 55, 61, 78, and 78%, respectively. QSM showed the highest overall importance among all contrasts for the correct prediction of the subcortical structures with “max_qsm” being the single highest factor of importance for the prediction task.

**TABLE 5:** Feature importance for each contrast and parameter.

	**General analysis of subcortical structures**				**Mean AoP**	**Functional group analysis**				**Mean AoP**
	**QSM**	**Na**	**MTR**	**T_1_**		**QSM**	**Na**	**MTR**	**T_1_**	
mean	0.044	**0.044**	0.020	0.038	**85%**	**0.068**	0.037	0.025	0.064	**81%**
var	0.026	0.026	**0.045**	0.034		0.016	0.033	0.024	0.052	
min	0.025	0.024	0.033	0.022		0.029	0.026	0.031	0.021	
max	**0.065**	0.042	0.016	0.025		0.037	0.028	0.015	0.013	
mean_grad	0.031	0.039	0.026	**0.039**		0.024	**0.063**	0.023	**0.070**	
var_grad	0.033	0.025	0.042	0.027		0.038	0.025	**0.042**	0.034	
min_grad	0.021	0.021	0.021	0.022		0.016	0.029	0.018	0.022	
max_grad	0.025	0.027	0.033	0.021		0.013	0.017	0.016	0.013	
Total	**0.273**	0.252	0.240	0.232		0.244	0.262	0.198	**0.294**	
	**(Epi-)Thalamic nuclei**					**Limbic nuclei**				
mean	**0.055**	**0.057**	0.020	0.019	**68%**	0.035	0.036	0.034	0.020	**90%**
var	0.038	0.025	0.043	0.025		0.015	0.018	**0.084**	0.019	
min	0.028	0.020	0.070	0.016		0.008	0.010	0.022	0.007	
max	0.049	0.021	0.014	**0.034**		**0.082**	0.042	0.010	0.058	
mean_grad	0.038	0.041	0.012	0.012		0.035	0.032	0.034	0.032	
var_grad	0.028	0.035	**0.056**	0.018		0.036	0.025	0.041	0.025	
min_grad	0.012	0.010	0.025	0.028		0.015	0.009	0.020	0.012	
max_grad	0.042	0.037	0.040	0.016		0.036	**0.056**	0.043	0.034	
Total	**0.294**	0.249	0.284	0.171		0.264	0.233	**0.291**	0.211	
	**ARAS**					**Extrapyramidal nuclei**				
mean	0.032	0.004	0.004	**0.043**	**100%**	0.029	0.018	0.011	0.024	**95%**
var	0.026	0.046	0.017	0.023		0.040	0.033	0.025	0.034	
min	0.026	0.031	0.010	0.023		0.024	0.027	**0.042**	0.032	
max_	**0.049**	**0.062**	**0.050**	0.021		**0.064**	**0.042**	0.020	0.028	
mean_grad	0.039	0.061	0.036	0.038		0.025	0.027	0.026	**0.039**	
var_grad	0.028	0.014	0.030	0.020		0.026	0.036	**0.042**	0.032	
min_grad	0.023	0.036	0.037	0.020		0.029	0.016	0.019	0.028	
max_grad	0.032	0.050	0.029	0.024		0.045	0.026	0.045	0.029	
Total	0.257	**0.309**	0.217	0.215		**0.287**	0.229	0.233	0.250	

*The mean test accuracy of prediction (mean AoP) is given in percent. The bold values indicate the most important feature.*

The overall accuracy of prediction for the functional groups was 81% (Figure 6B) with the highest accuracy of 96% achieved for fiber tracts followed by an accuracy of 86% for extrapyramidal and 83% for (epi-)thalamic nuclei. For the functional group prediction, the T_1_ sequence had the highest overall importance out of the four sequences. The highest single importance was achieved by the feature “mean_grad_T_1_.”

Overall accuracy of prediction within each of the functional groups varied strongly (Figures 6C–G and Table 5). Within the (epi-)thalamic group of nuclei the overall accuracy of prediction was 68% and while LGB and Pul were correctly predicted with 80 and 82%, respectively, the MGB was often confounded with the LGB and correct prediction was only achieved in 46%. In the group of limbic nuclei, the overall accuracy correct prediction was 90% with an excellent accuracy for the VP of 100% and an accuracy of 85, 88, and 86% for the BNST, the NAC and the NBM, respectively. The nuclei affiliated with the ARAS show an overall accuracy of prediction of 100% with excellent differentiation of DRN, PPN and VTA. In the extrapyramidal system, all structures were classified with an accuracy of 95%. Accuracy of prediction was lowest for the substantia nigra with 90%.

## Discussion

Subcortical structures have initially been defined by anatomy and histology-based atlases (Lemaire et al., 2019) and were further characterized in vivo by volumetric imaging approaches (Summerfield et al., 2005; Dickson, 2012; Shams et al., 2017). While recent advances in quantitative MRI increasingly enable the detection of disease-associated MR signal alterations in subcortical nuclei (Cassidy et al., 2019), the predominant mono-parametric approaches yield varying disease specificities (Saeed et al., 2017). To investigate a multimodal approach for characterization of subcortical nuclei, this study explored a combined analysis of quantitative MR values of four different MR contrasts in subcortical structures of healthy volunteers. Only considering multi-parametric voxel-intrinsic information from each VOI, the combination of these MR contrasts allowed for good differentiability of nuclei in the ARAS as well as in the limbic and extrapyramidal system. Even across all subcortical structures, the MR value-based random forest analysis reached an overall prediction accuracy of 85%, demonstrating that multi-parametric quantitative MRI enables a distinction of subtle histoarchitectural tissue differences within subcortical structures based on voxel-intrinsic MR values. The differentiability of cortical cerebral structures and white matter tracts based on multi-parametric voxel-intrinsic information is a focus of current research and has been shown feasible to a certain extent (German et al., 2021). The presented study focuses on subcortical structures and shows that multi-parametric quantitative MRI is able to distinguish subtle histoarchitectural tissue differences within subcortical nuclei in healthy volunteers for the first time. This histoarchitectural differentiability of subcortical structures may itself be the requisite for the MR-based discrimination of subcortical diseases in the future as protein depositions in characteristic combinations of subcortical cerebral nuclei in neurodegenerative diseases (Dugger and Dickson, 2017) may induce a loss of MR-contrast based differentiability between subcortical structures. A future application of this approach in clinical studies will strongly benefit from an automated segmentation of subcortical structures as a manual delineation is not readily feasible in larger cohorts or multi-center studies. As recent advances regarding the automated segmentation of subcortical structures due to quantitative and multi-contrast MRI techniques (Bazin et al., 2020; Corona et al., 2020) help to increase the number of visible subcortical nuclei (Xiao et al., 2016; Keuken et al., 2018; Najdenovska et al., 2019) and quantitative multi-contrast MRI enables excellent automated distinguishability between subcortical nuclei and the surrounding tissue (Supplementary Figures 2, 3), synergies with segmentation approaches are very likely.

Regarding the characterization of specific subcortical nuclei, the integrated multimodal visualization of quantitative MR values in each structure agreed with the machine learning-based analysis, as both yielded a less successful classification only within the group of (epi-) thalamic nuclei. However, while the differentiation between the BNST, NBM, and NAC in the group of limbic nuclei seems difficult based on integrated multimodal visualization (Figure 5B), the machine learning-based analysis still reached an accuracy of correct prediction between 84 and 89%. Similarly, the machine learning-based discrimination of extrapyramidal nuclei was excellent in spite of considerable overlap between NC and Pal, SN and Pal as well as RN and STN in Figure 5D, illustrating a methodical advantage. One reason for this advantage may be the larger number of features in the machine learning-based approach. While visual or quantitative, integrated multimodal visualization analysis of minima, maxima, mean and possibly variance may be feasible, unaided comparison of gradients representing heterogeneity within the different structures remains challenging, although varying distribution of iron in structures such as the STN has been described to cause a characteristic heterogeneity in susceptibility imaging (Dormont et al., 2004). However, for automated pattern recognition, gradient analysis is well feasible and widely accepted in image analysis (Canny, 1986). The prominence of the “mean_grad_T_1_” feature as the single highest factor of importance for the prediction of functional group affiliation in this study furthermore underlines the importance of gradient features. The random forest-based machine learning approach was applied to quantify multiparametric classification of subcortical nuclei as it is not prone to overfitting and most importantly allows for the assessment of feature contribution to the classification performance enabling an understanding of the specific influence of features and contrasts on the prediction task (Kleinberg, 1990). Other common machine learning models were considered, showing that linear models such as linear discriminant analysis (LDA) and logistic regression are not powerful enough to reach good performance and neural networks or support vector machines (SVM) depend strongly on hyperparameter fine tuning and tend to overfitting, thus leading to a less accurate performance (Supplementary Figure 1).

Focusing on the importance of each contrast for the classification analysis, susceptibility values were demonstrated to be the single most important factor for the classification of the subcortical structures in general; however, the other contrasts seem only minimally less important (Table 5). The importance of multiple contrasts for structure differentiation is underlined by the finding that the MGB, NAC, LGB, NC, and DRN share comparatively high sodium concentrations, low MTR and long T_1_ times and were the most often incorrectly classified structures mainly due to confusion between one another although susceptibility values differed broadly among them (Figure 3).

The four specific MR contrasts applied in this study were chosen to provide a maximally complementary combination of quantitative MR values and strong tissue contrast for delineation of subcortical nuclei. While QSM provides both optimal demarcation of subcortical structures and quantitative assessment of susceptibility (Deistung et al., 2013b), sodium imaging enables an estimation of total sodium concentrations without being markedly influenced by changes in susceptibility (Nagel et al., 2009; Schneider et al., 2018), and MTR correlates with tissue concentrations of macromolecules (Henkelman et al., 2001; Horsfield et al., 2003; Peper et al., 2013). PD-weighted imaging and T_1_ maps additionally enhance visualization of subcortical structures with lower iron content (Straub et al., 2019), and the overlay of MT-saturated PD and QSM enables increased delineation of the BNST, PPN, DRN, and ML compared to QSM alone (Figure 2).

The chosen MR contrasts furthermore complement one another well, as the performed voxel-wise signal analysis displayed moderate correlations only between T_1_ and sodium signal, and between T_1_ and MTR. These correlations can be explained by the known correlations of T_1_ time and sodium imaging to extracellular volume (ECV) (Jakobsen et al., 1995; Madelin and Regatte, 2013; Madelin et al., 2014). An ECV-associated increase in the mobile proton pool is associated with a decrease in MTR values and can explain the inverse correlation of MTR and T_1_ times (Laule et al., 2003). However, only a weak correlation is displayed between sodium imaging and MTR, possibly due to the latter’s additional dependence on the exchange rate between proton pools. Finally, especially iron rich structures such as the SN, STN, RN, Pal, and VP show shortened T_1_ times, and the demonstrated weakly negative correlation of T_1_ times and QSM is in support of a previously described, faster longitudinal relaxation in the presence of iron (Trujillo et al., 2017). The weakness of this correlation may be due to the influence of substances like neuromelanin in the VTA and SN that are known to further shorten T_1_ times (Trujillo et al., 2017). The similar T_1_ values in VTA and SN may underline a similar quantity and configuration of neuromelanins in the two nuclei while high additional iron deposit in the form of ferritin mainly in the SN (Hare et al., 2014) may result in a good discrimination of these two structures on QSM.

So far, quantitative values for the MR contrasts applied here have only been reported for a limited number of subcortical structures, and a comparison of measured values is therefore limited. Absolute mean values for susceptibilities as well as T_1_ times measured in the Pal, Put, CN, SN, and RN are comparable to previously reported susceptibilities and T_1_ relaxation times at 7T (Deistung et al., 2013b; Lim et al., 2013; Straub et al., 2019). However, regarding MTR, only the relation between MTR values within the different subcortical structures is in agreement with other studies due to differing sequence parameters (Filippi et al., 2001; Helms et al., 2008). Absolute mean sodium concentrations have been reported by Ridley et al. for the Pal, thalamic structures, Put and CN at 28 ± 9.7% below the concentrations measured in this study (Ridley et al., 2018). However, in contrast to the sodium concentrations reported here, sodium concentrations were referenced with an external agar-filled cylinder and recorded at a lower nominal isotropic resolution of 3.5 mm. Interestingly, comparison of sodium measurements with histology-based cellular densities in the basal ganglia published by Salvesen et al. (2015) revealed a strong positive correlation of sodium measurements in the STN, PU, CN, Pal, and RN with total cell densities or neuron densities for the first time (*r*_Pearson_ = 0.9, *p* < 0.05). At the same time a weakly negative and not statistically significant correlation was found between sodium measurements in these structures and oligodendrocyte densities (*r*_Pearson_ = −0.2, *p* = 0.74). Reduced sodium levels have thus far been thought to be an indicator of high cellular density and reduced ECV as previous neurooncological studies on glial tumors have shown sodium concentrations to correlate with apparent diffusion coefficient (ADC) values (Schepkin et al., 2005; Madelin et al., 2014). Likewise, sodium concentrations increased in cerebral gray matter of patients with neurodegenerative disease (Mellon et al., 2009; Kim et al., 2017). However, possibly due to a histoarchitectural, cell type associated relative increase in ECV with increasing neuron density, the findings in this study suggest that this perspective cannot be transferred to physiological comparisons of cell densities among subcortical nuclei.

This study has several limitations: The relatively low number of studied subjects and the focus on a young age group limits a generalization of measured quantitative values and limits the training of machine learning algorithms. A limitation for the analysis of the integrated multimodal visualization of quantitative MR values as well as the machine learning-based analysis are partial volume effects that bias measurements especially in small subcortical structures. This effect is particularly pronounced for low image resolutions as used for sodium imaging; consequently, MB, LC, and HB that lie immediately adjacent to CSF have been excluded from the machine learning-based analysis to limit partial volume bias.

Moreover, no dedicated *B*_0_ and *B*_1_ correction or correction for gradient delays was performed. Susceptibility mapping has been shown not to depend on *B*_1_inhomogeneties even in multi-center ultra-high field studies QSM (Rua et al., 2020; Voelker et al., 2021), but the correction of dynamic *B*_0_ fluctuations improves image quality in susceptibility-based methods and could thus enable a better characterization for multi-contrast approaches including QSM segmentation (Jorge et al., 2020). However, MTR measurements are more sensitive to *B*_1_ inhomogeneites and especially for multi-site studies, *B*_1_ correction would be advisable (Barker et al., 2005). For sodium imaging there are first studies in which a correction of the gradient trajectory was used (Lu et al., 2010), however twisted projection pulse sequences were used instead of a radial trajectory. In the future, corrections for gradient delays and *B*_0_/*B*_1_-inhomogeneties might be implemented to improve the quantitative accuracy of sodium MRI (Lommen et al., 2016; Gerhalter et al., 2020), especially to improve the comparability of data in multi-center studies.

An even larger number of MR contrasts could further enhance quantitative MR value-based differentiability of subcortical structures. As multi-echo gradient echo data were acquired in this study, it would have been also possible to calculate $T_{2}^{*}$ or $R_{2}^{*}$ maps, however, good correlations of QSM and $R_{2}^{*}$ relaxation rates have been observed especially in iron rich subcortical nuclei (Wang et al., 2017) and the additional use of $R_{2}^{*}$ relaxation rates would have undermined the aim to use relatively independent MR contrasts. Finally, although many UHF studies that include QSM only use three echos (Deistung et al., 2013b; Straub et al., 2019), as done here, or even only a single echo (Mattern et al., 2019), it is recommended to use a high number of echoes for better signal- and contrast-to-noise ratios depending on the intended application (Haacke et al., 2015).

## Conclusion

The combination of quantitative MR values from quantitative multiparametric MRI, namely QSM, sodium imaging, MTR and T_1_ mapping, enabled excellent characterization and differentiation of subcortical nuclei of the ARAS as well as the limbic and extrapyramidal system based on subtle contrast differences between tissues in these subcortical nuclei. Multiparametric quantitative MRI may thus be sensitive enough to enable the detection and specification of pathologic histoarchitectural tissue changes in subcortical nuclei in the future.

## Data Availability Statement

The raw data supporting the conclusions of this article will be made available upon reasonable request.

## Ethics Statement

The studies involving human participants were reviewed and approved by the Institutional Ethics Committee at the University of Heidelberg. The participants provided their written informed consent to participate in this study.

## Author Contributions

TS and SS were central for the conception of this study. JM and PW were instrumental regarding machine learning. All authors contributed to the compilation of the manuscript.

## Conflict of Interest

The authors declare that the research was conducted in the absence of any commercial or financial relationships that could be construed as a potential conflict of interest.
