# Supplementary material for: Multiparametric MRI for Characterization of the Basal Ganglia and the Midbrain
Source: Front Neurosci. 2021 Jun 21;15:661504. doi: 10.3389/fnins.2021.661504 (PMC8255625; doi:10.3389/fnins.2021.661504)
Supplement: Supplementary file 1 [file Table_1.DOCX]

**Supplementary material**

1. **Comparison with different methods**

Besides random forests, there are many other possible model choices. Since the number of samples per class in this study was small, self-regularizing ensemble methods such as random forests performed best and most stable across most of the analyzed settings (varying the number of classes), except for predicting functional group of each VOI (i.e. multiple samples per class), where also a neural network (two layers with 100 neurons each with ReLU activations) and a support vector machine (SVM with Gaussian kernel) achieve comparable results. In summary, the results of this experiment are shown as multiple bar plots (Figure S1), where for each setting (all 18 classes; 12 and 6 random classes sampled out of all classes for each fit and five functional groups as in Figure 6b), each model under consideration is compared. In addition to random forest, neural network and SVMs, linear models such as linear discriminant analysis (LDA) and logistic regression (generalization of linear regression to classification) were also considered. For each experiment 100 runs of five-fold cross-validation were conducted, and the mean accuracy and standard deviation (as black error bars) is reported. The gray bars represent the number of samples for each setting, showing that random forests perform best in comparison to the other models (for 18,12 and 6 classes), except for the case of predicting functional groups, where neural network and SVM achieve better results, which is expected due to the property of sample efficiency. Nevertheless, random forests stay within the error bars and are comparable in terms of performance.


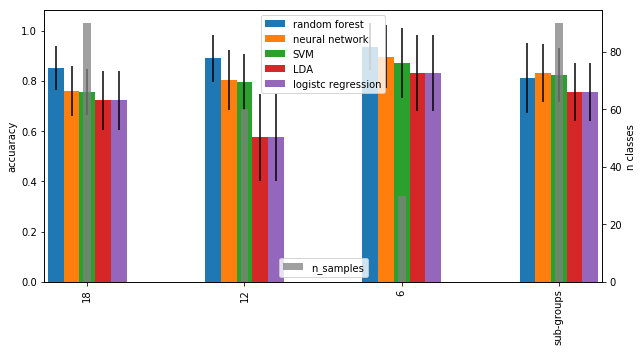


Figure S1

1. **Discimination analysis against background**

To study the discrimination ability not just between subcortical structures themselves, but also across other cerebral tissues, a binary classification experiment was conducted, in which all defined subcortical structures were treated as one common class for nuclei. Based on the segmentation map, the complementary voxels were used within a bounding box as background class (including all other tissues apart from nuclei), where the bounding box was the smallest volume containing all segmented voxels (Figure S2). This was also tested for smaller bounding boxes (centered around center of mass of segmentation maps) to decrease the variability of considered tissues, but in all cases a near perfect performance was observed (also across models as considered above) (Figure S3).

c
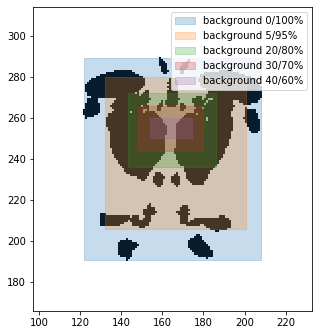


Figure S2


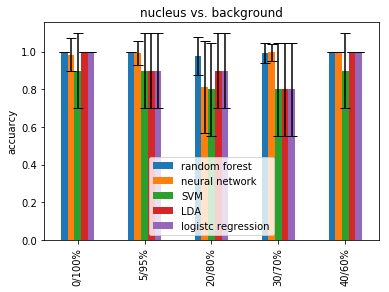


Figure S3
